# The art and science of head nerve blocks in urgent care settings

**DOI:** 10.1186/s12245-026-01362-3

**Published:** 2026-09-09

**Authors:** Zoë M. Rushetsky, Grace E. Lynch, Bhavik Singh, Ambika Jokhulall, Donald Penney

**Affiliations:** Philadelphia College of Osteopathic Medicine, 625 Old Peachtree Rd NW, Suwanee, GA 30024 USA

**Keywords:** Scalp nerve block, Regional anesthesia, Laceration repair, Head and neck injuries

## Abstract

The purpose of this review is to describe the role of regional scalp nerve blocks as an alternative to direct local infiltration for head and scalp laceration repair in emergency and urgent care settings, where tissue swelling from infiltration can obscure landmarks and complicate wound closure. Methods included a narrative review of published studies and procedural descriptions focusing on scalp sensory anatomy, nerve block techniques, anesthetic selection, and safety considerations, along with the development of simplified illustrations and surface-landmark images tailored to outpatient clinicians. Results demonstrate that regional scalp blocks reliably provide effective anesthesia with less tissue distortion and lower anesthetic volume than infiltration, yet existing resources are often highly technical or dispersed across subspecialty literature. The diagrams created for this review consolidate essential anatomic and procedural information into a format that is easier to reference during clinical care and illustrate how a small number of targeted injections can anesthetize common laceration patterns involving the forehead, temporal region, and occiput. The conclusion is that regional scalp nerve blocks are safe, efficient, and less frequently incorporated into routine emergency practice for selected head and scalp injuries, and that combining current evidence with practical visual guides may increase clinician confidence and adoption in urgent care environments.

## Introduction

**Fig. 1:**
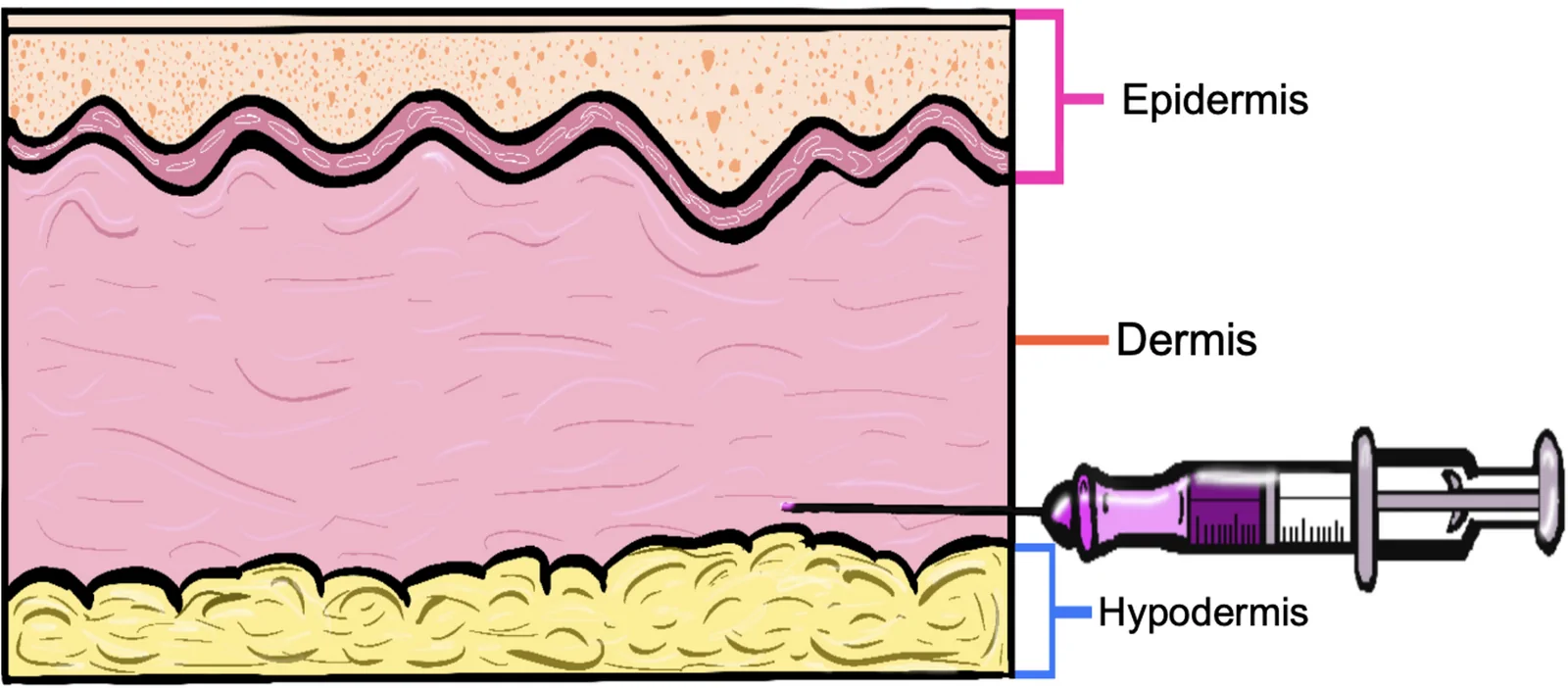
Local infiltration anesthesia is administered in the tissue plane immediately below the dermis, at the interface with the hypodermis. Illustrated by: Zoë Rushetsky

Head and neck injuries such as facial fractures and scalp lacerations are a routine challenge amongst children and elderly in an urgent care setting. Lacerations requiring closure account for approximately 4.8% of all ED visits in the United States, with 38% of those being of the face and scalp [1]. The average age of affected patients is 30-years-old, however, children are disproportionately affected with up to 42% of cases occurring in ages 0–17 years of age. Males are disproportionately affected, accounting for more than half of all reported cases [2]. These injuries frequently result from physical assault, falls, contact sports, motor vehicle collisions, or animal bites. For years, anesthesia was most often managed by injecting anesthetic directly into the wound as shown in Fig. 1.

**Fig. 2:**
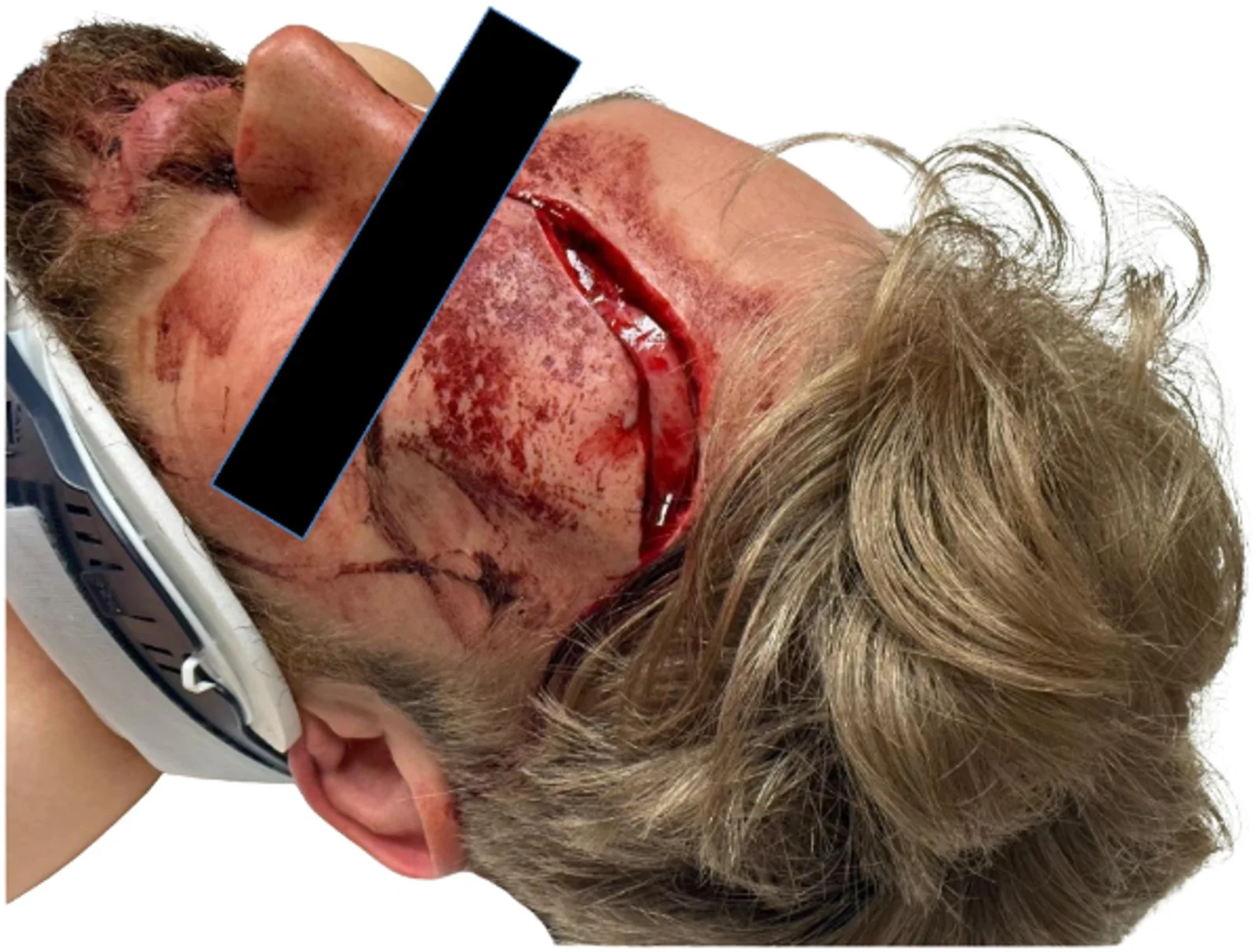
Clinical image of extensive deep laceration of the left frontotemporal region, illustrating injury patterns relevant to regional anesthesia and wound management in acute trauma

**Fig. 3:**
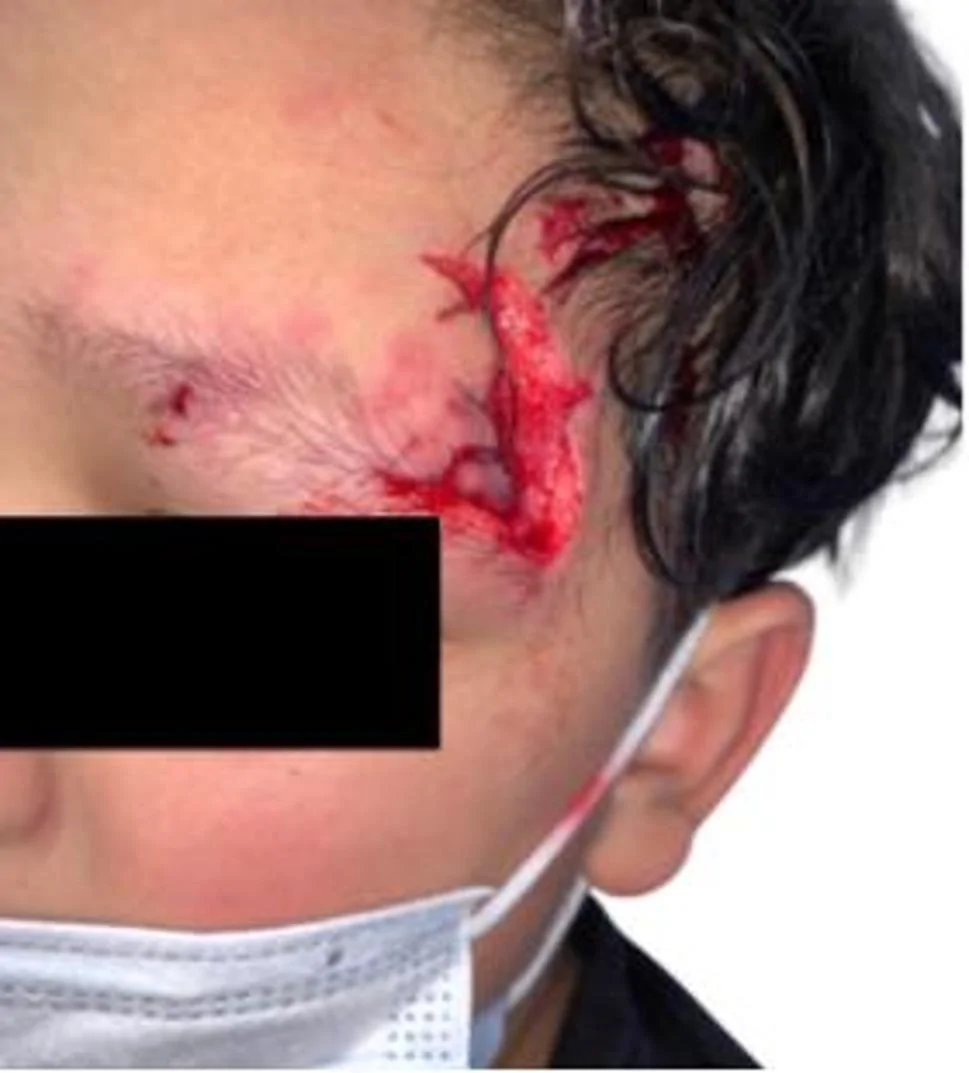
Clinical image showing a deep, irregular laceration of the left temporal-preauricular region sustained after a dog bite

Direct infiltration of local anesthetic has traditionally been the standard approach for scalp laceration repair (Fig. 1). However, tissue distortion from injected anesthetic can obscure wound anatomy, separate tissue planes, and complicate repair. Regional scalp nerve blocks avoid these limitations while providing effective analgesia [3, 4]. Although routinely used in neurosurgery, particularly during awake craniotomies, they remain underutilized in urgent care despite being straightforward to perform and reducing the need for sedation and multiple local injections [3, 4]. Clinical examples of deep scalp lacerations that would benefit from regional anesthesia are shown in Figs. 2 and 3.

### Scalp sensory nerve anatomy

**Fig. 4:**
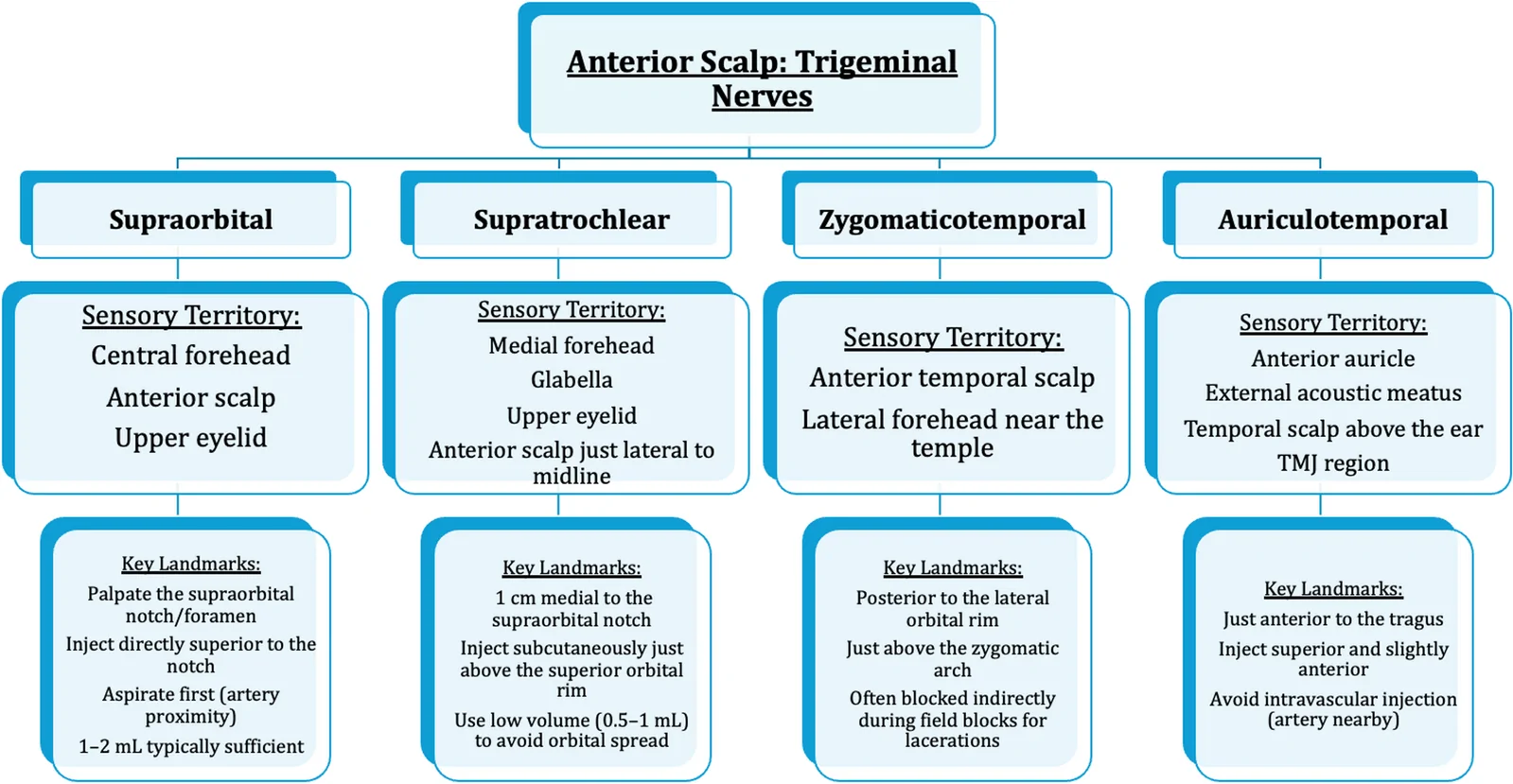
Summary of anterior scalp sensory innervation by trigeminal nerve branches, including sensory territories and key surface landmarks relevant to regional nerve blocks

A clear understanding of the scalp’s sensory nerve anatomy is essential for performing effective and safe regional nerve blocks. Sensory innervation of the scalp is supplied by branches of both the trigeminal nerve anteriorly and the cervical plexus posteriorly [5]. An overview of the anterior scalp sensory nerves, their corresponding sensory territories, and key surface landmarks relevant to regional anesthesia is shown in Fig. 4.

**Fig. 5:**
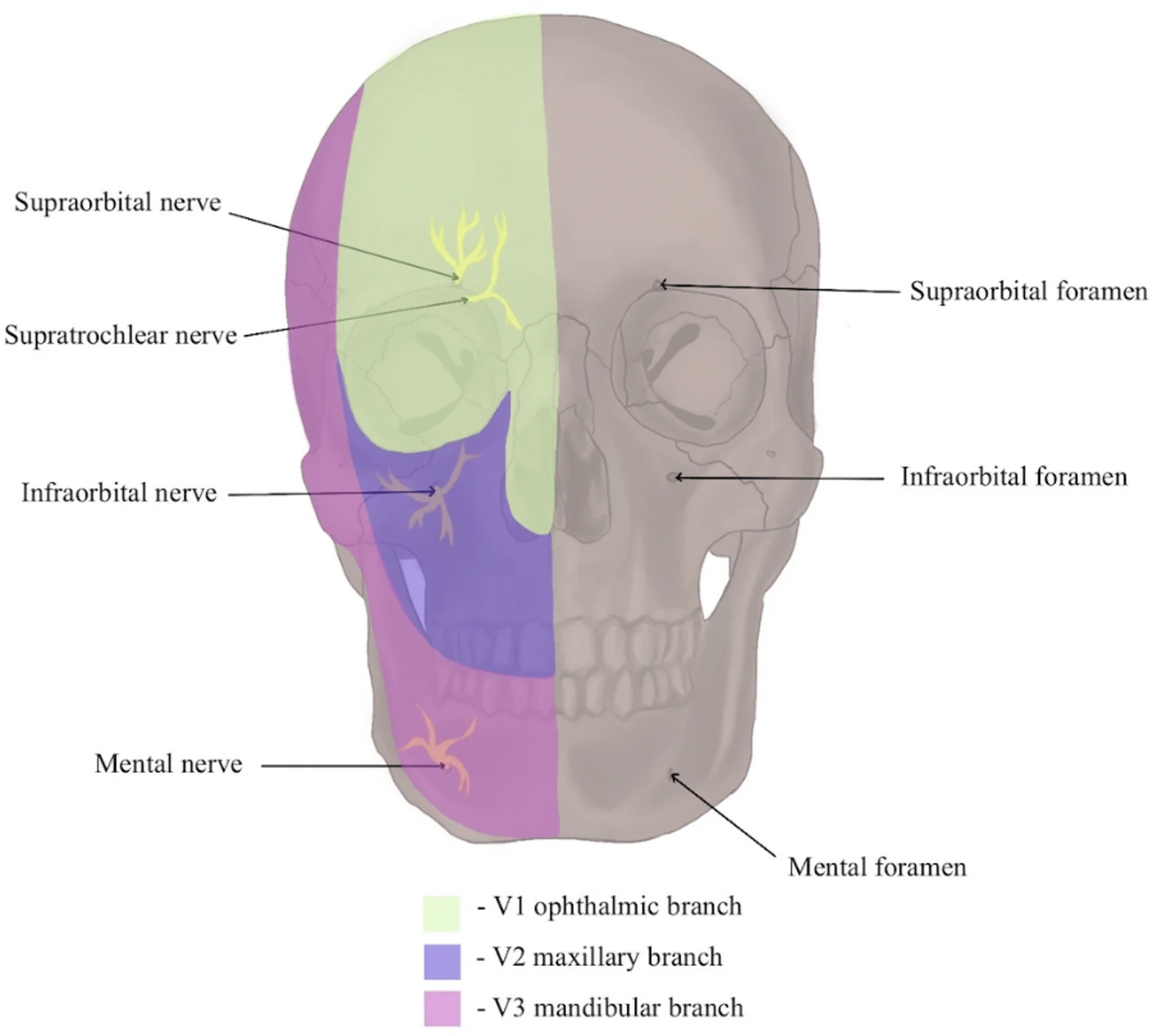
Anterior-posterior view of the anatomy of the supraorbital nerve, supratrochlear nerve, infraorbital nerve, mental nerve. The colored overlay represents the cutaneous sensory territories of the ophthalmic (V1), maxillary (V2), and mandibular (V3) divisions of the trigeminal nerve. Illustrated by: Ambika Jokhulall

**Fig. 6:**
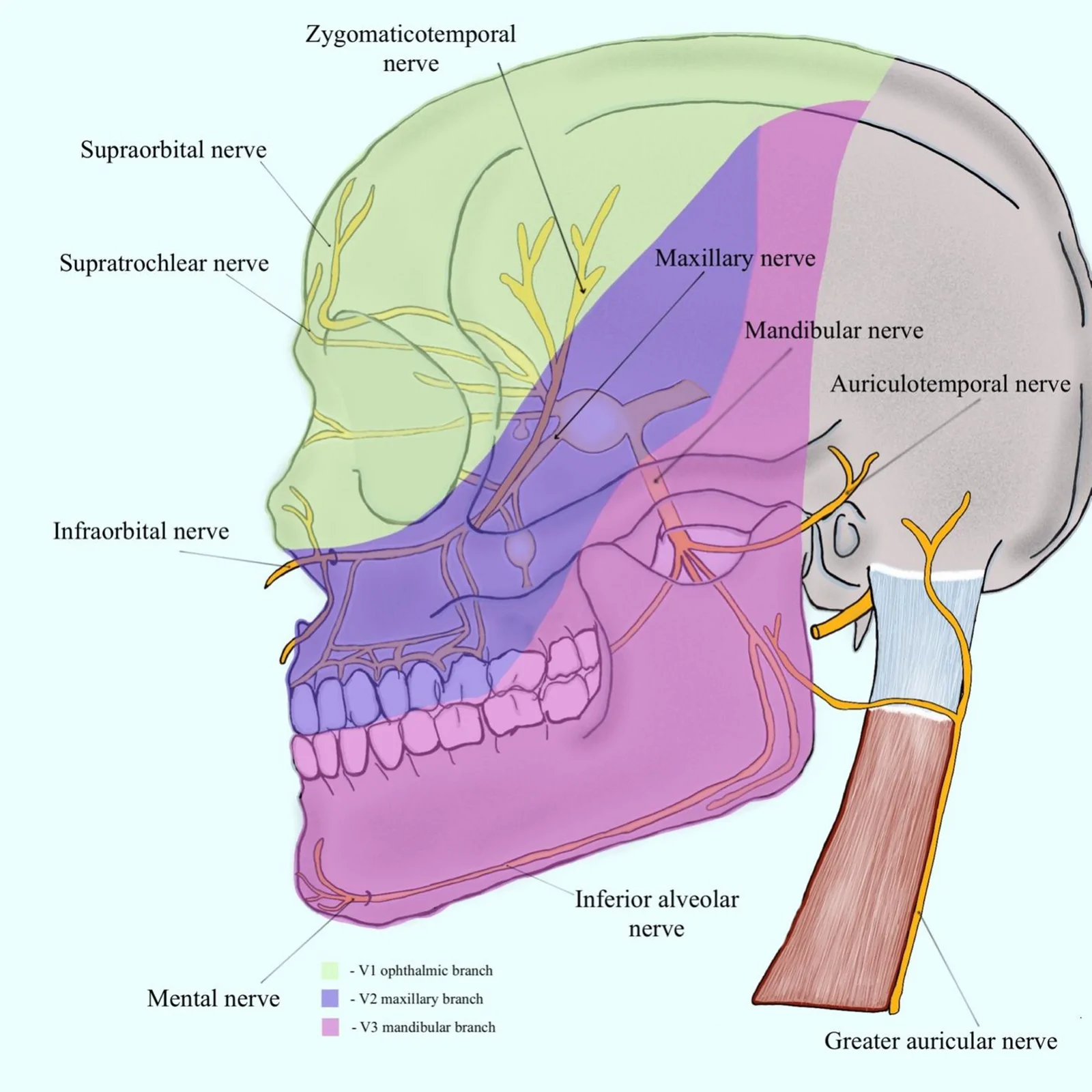
Lateral view illustrating the anatomical course of the supraorbital, supratrochlear, infraorbital, mental, maxillary, mandibular, auriculotemporal, greater auricular, zygomaticotemporal, and inferior alveolar nerves with their corresponding cutaneous sensory distributions. The colored overlay represents the sensory territories of the ophthalmic (V1), maxillary (V2), and mandibular (V3) divisions of the trigeminal nerve. Illustrated by: Zoë Rushetsky

Anteriorly, the supratrochlear and supraorbital nerves, both branches of the ophthalmic division (V1) of the trigeminal nerve, exit the superior orbital rim above the eyebrow [5]. These nerves provide sensation to the upper eyelid, forehead, and anterior scalp extending posteriorly to the coronal suture [6]. Detailed anterior and lateral views of the craniofacial nerve anatomy are illustrated in Figs. 5 and 6.

### General consideration for scalp nerve blocks

Prior to performing a scalp nerve block, informed consent should be obtained and contraindications assessed. Contraindications include patient refusal, allergy to local anesthetics, infection or cellulitis at the injection site, and inability to identify anatomic landmarks. Relative contraindications include coagulopathy or therapeutic anticoagulation, which may increase the risk of bleeding or hematoma formation. Patients should be monitored using standard ASA monitors, including pulse oximetry, noninvasive blood pressure, and electrocardiography, throughout the procedure and observed afterward for signs of local anesthetic systemic toxicity (LAST). The skin should be prepared with chlorhexidine solution while avoiding ocular exposure, and standard sterile precautions, including gloves, mask, and cap, should be used. Careful aspiration before injection is recommended to reduce the risk of inadvertent intravascular administration [7, 9]. If adequate anesthesia is not achieved after the initial scalp nerve block, the provider should reassess the distribution of the wound and consider whether or not additional sensory nerves require blockage, whether it is due to inaccurate localization or overlapping innervation. In some cases, supplemental local anesthetic infiltration along the wound margins may be performed as needed to achieve complete analgesia while remaining within the recommended maximum dosing limits. In the cases of more complex lacerations, targeted scalp nerve blocks may also be combined with partial ring blocks to provide additional anesthetic coverage.

### Blocks for the anterior scalp

The patient is positioned supine, and the supraorbital notch is palpated along the superior orbital rim in line with the mid-pupil when the patient is looking straight ahead. A 25- to 27-gauge needle is inserted just superior to the supraorbital rim perpendicular to the underlying bone until gentle contact with bone is appreciated. The needle is then withdrawn slightly before aspiration and injection of 1–3 mL of local anesthetic. The injection is extended medially to anesthetize the supratrochlear nerve. Care should be taken to avoid entering the supraorbital foramen or directing the needle toward the orbit to minimize the risk of ocular injury [5–7].

The supratrochlear nerve is located medially near the inner aspect of the eyebrow, whereas the supraorbital nerve emerges from the supraorbital notch or foramen approximately 2.5 cm lateral to the midline [5]. These nerves provide sensation to the upper eyelid, forehead, and anterior scalp to the coronal suture [6]. Indications for these nerve blocks include procedures requiring anesthesia of the forehead extending to the vertex of the scalp [7].

**Fig. 7:**
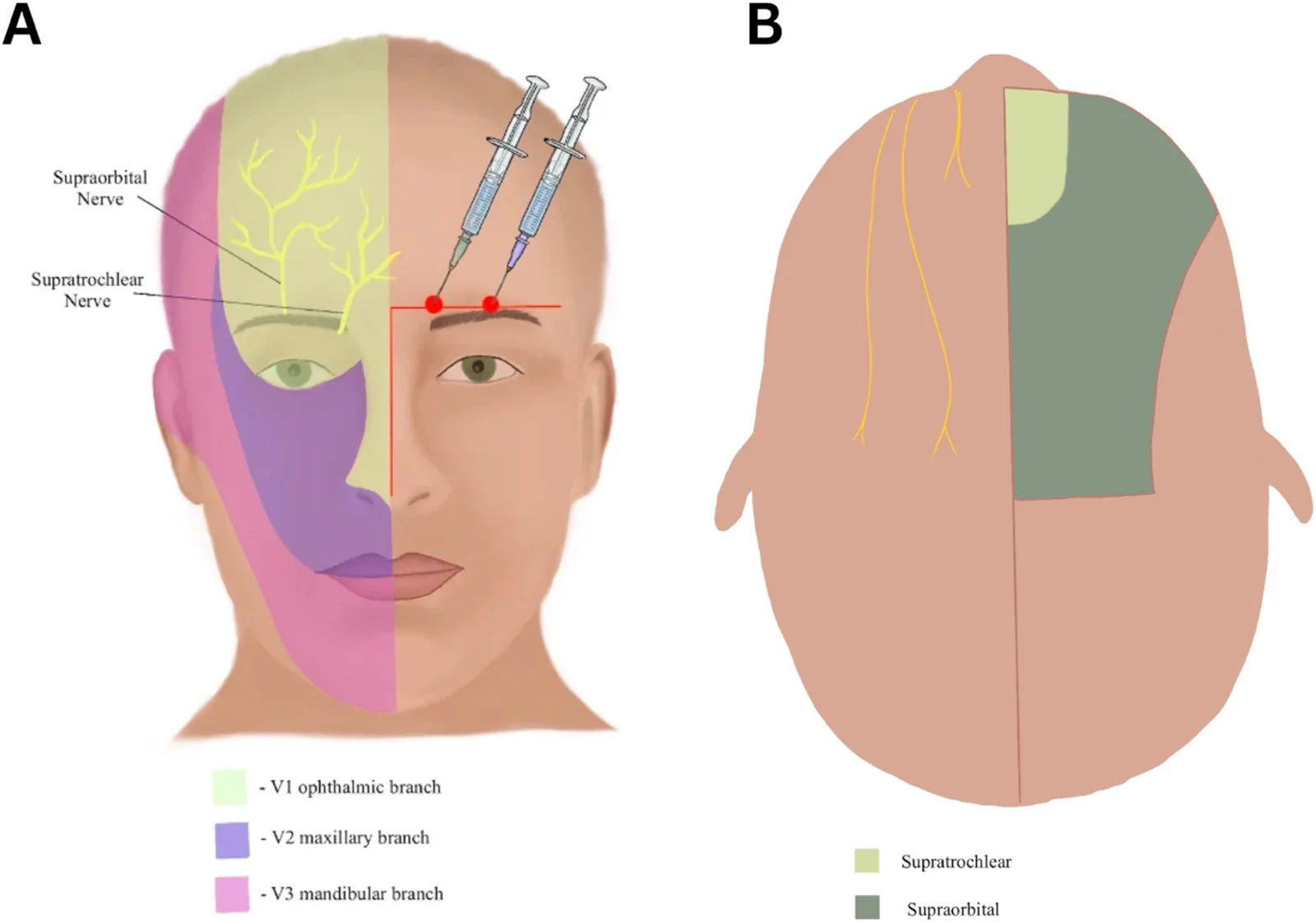
**(A)** Surface anatomy, cutaneous sensory distribution, and injection sites for the supraorbital and supratrochlear nerve blocks. The colored overlay depicts the sensory territories of the ophthalmic (V1), maxillary (V2), and mandibular (V3) divisions of the trigeminal nerve, with the supraorbital and supratrochlear nerves arising from the ophthalmic division (V1). The green needle denotes the supratrochlear nerve injection site, and the purple needle denotes the supraorbital nerve injection site. **(B)** Superior view of the scalp illustrating the cutaneous sensory territories supplied by the supraorbital and supratrochlear nerves. Illustrated by: Ambika Jokhulall

Blocking the supraorbital and supratrochlear nerves would provide regional anesthesia with two injections reducing the need for multiple injections in the wound and the amount of local anesthetic utilized. The injection site and anesthetized region for these nerves are shown in Fig. 7.

### Blocks for the lateral scalp

The lateral scalp receives input from the zygomaticotemporal and auriculotemporal nerves, which arise from the maxillary (V2) and mandibular (V3) branches of the trigeminal nerve, respectively [8].

The zygomaticotemporal nerve exits just above the zygomatic arch, approximately 1 cm posterior to the lateral orbital rim. Here, a fan-shaped injection of 1–2 mL local anesthetic into the subdermal layer provides effective coverage. The zygomaticotemporal nerve exits just superior to the zygomatic arch near the lateral orbital rim. The needle is inserted anteriorly toward the posterior aspect of the lateral orbital rim, then advanced inferiorly along the concavity of the lateral orbital rim to the level of the lateral canthus. Local anesthetic (1–2 mL) is injected in a fan-shaped fashion to both the superficial (subdermal) and deep tissue planes while avoiding the orbit to minimize the risk of ocular injury [8, 9].

**Fig. 8:**
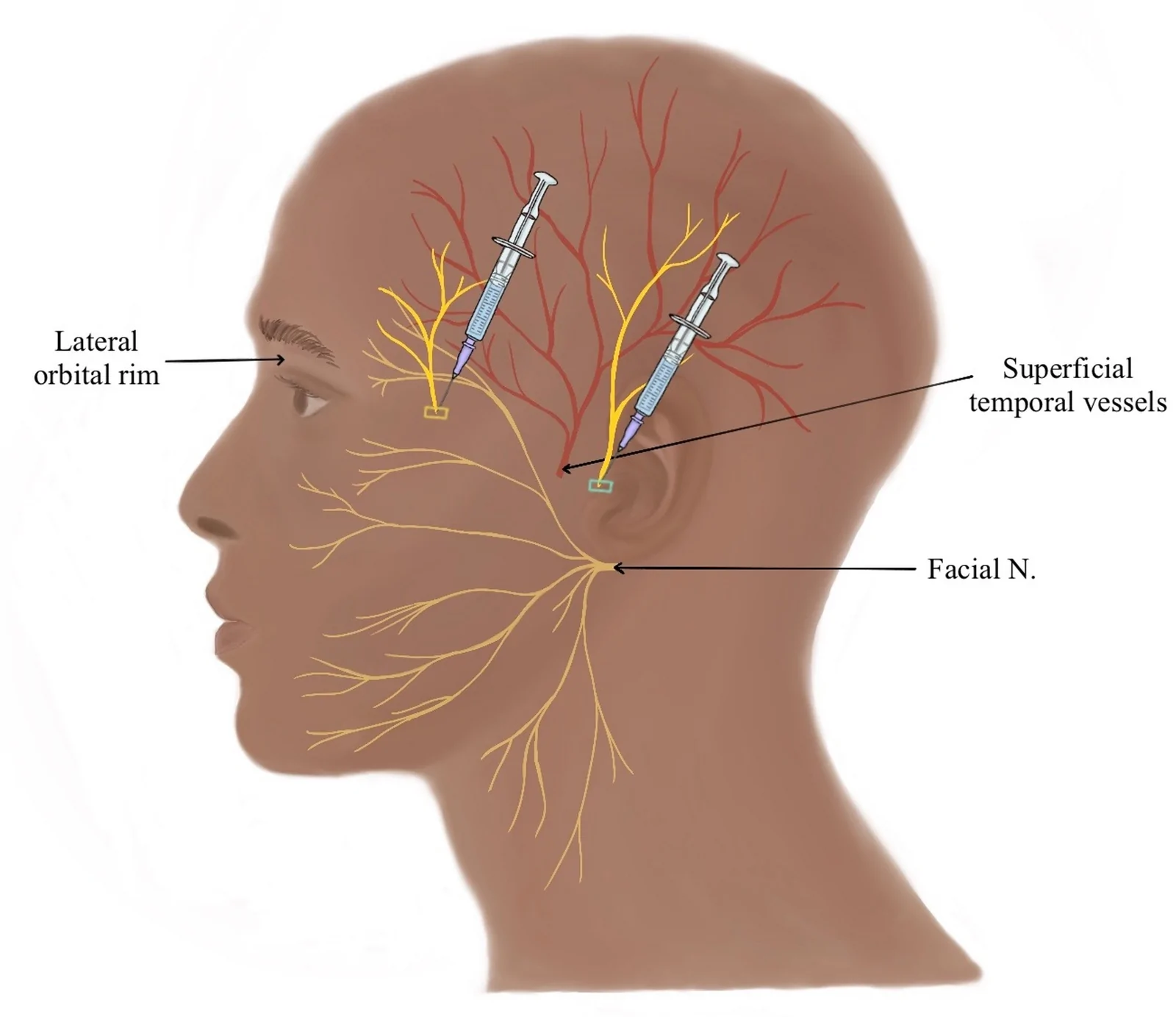
Injection sites for the auriculotemporal and zygomaticotemporal nerve blocks. The blue rectangle indicates the auriculotemporal nerve block location anterior to the tragus, and the yellow rectangle indicates the zygomaticotemporal nerve block location near the lateral orbital rim. The syringes illustrate the direction of local anesthetic infiltration. The superficial temporal vessels are shown as an important vascular landmark to identify and avoid during injection. The facial nerve is included to illustrate its proximity to the auriculotemporal injection site and to emphasize the importance of the more posterior injection approach in minimizing the risk of inadvertent facial nerve blockade. Illustrated by: Ambika Jokhulall

The auriculotemporal nerve travels 1–1.5 cm anterior to the superior border of the pinna, where it courses just posterior to the superficial temporal artery, which serves as an important landmark. The superficial temporal artery should first be palpated to identify its pulse. The needle is then inserted just posterior to the artery, and 1–2 mL of local anesthetic is injected after careful aspiration to minimize the risk of intravascular injection. This location is preferred over the more anterior pretragal approach, as deep injection approximately 1.5 cm anterior to the tragus may result in unintended facial nerve blockade [8, 9]. Injection sites and relevant surface landmarks for these nerve blocks are shown in Fig. 8.

### Blocks for the posterior scalp

**Fig. 9:**
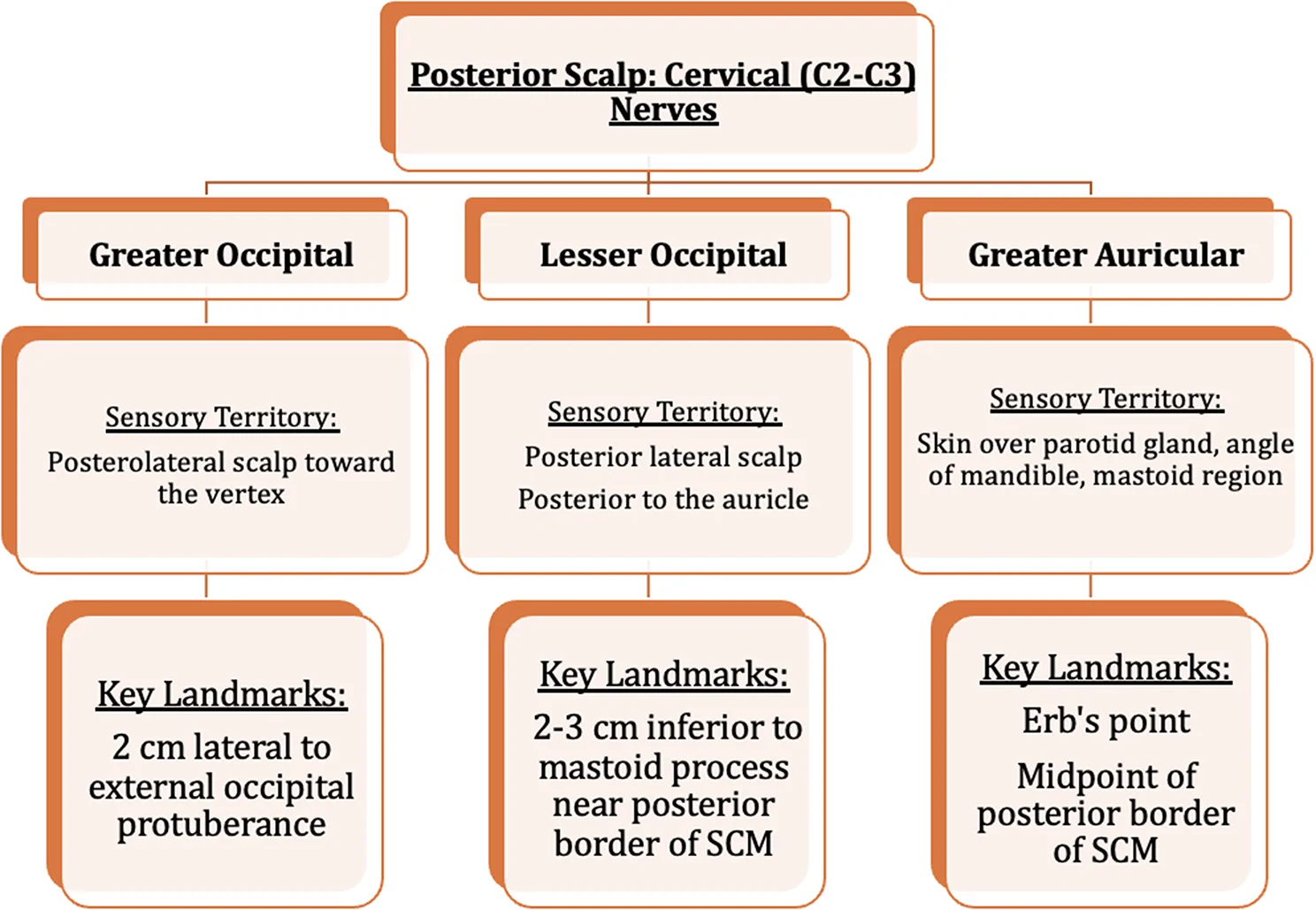
Summary of posterior scalp sensory innervation from cervical (C2–C3) nerves, highlighting sensory territories and surface landmarks used to guide regional anesthesia

Posterior scalp sensation is supplied primarily by branches of the cervical plexus (C2–C3). A summary of the posterior scalp sensory nerves, their sensory territories, and key surface landmarks is shown in Fig. 9.

The greater occipital nerve, arising from the dorsal ramus of C2, emerges approximately 2 cm lateral to the external occipital protuberance and is typically located just medial to the occipital artery, which serves as an important landmark for localization. Care should be taken to identify and avoid the occipital artery during injection to minimize the risk of inadvertent intravascular injection. A subcutaneous injection of 1–2 mL of local anesthetic at this location provides reliable anesthesia of the posterior scalp within the greater occipital nerve distribution.

The lesser occipital nerve, originating from the cervical plexus (C2–C3), ascends toward the mastoid process along the posterior border of the sternocleidomastoid muscle. When anesthesia of the posterolateral scalp is required, local anesthetic may be infiltrated subcutaneously along the superior nuchal line between the mastoid process and the external occipital protuberance to achieve blockade of the lesser occipital nerve [8].

The greater auricular nerve, another branch of the cervical plexus (C2–C3), provides sensation to the skin over the mastoid region, parotid gland, lower auricle, and angle of the mandible. For procedures involving the posterior auricular or mastoid region, subcutaneous infiltration along the superior nuchal line from the mastoid process toward the external occipital protuberance may simultaneously anesthetize the greater auricular, lesser occipital, and greater occipital nerves while avoiding unnecessary blockade of the transverse cervical and supraclavicular nerves [8].

**Fig. 10:**
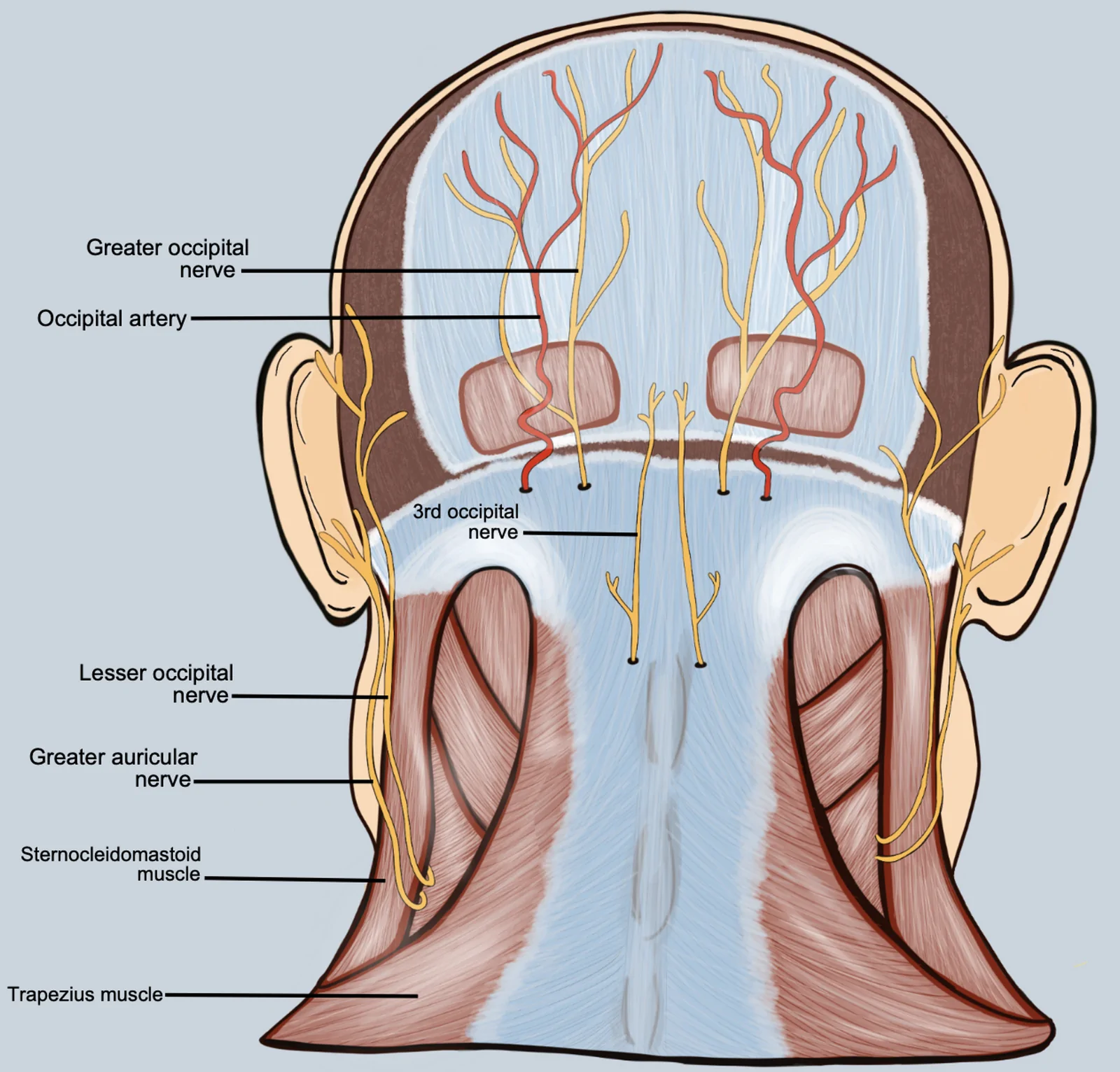
Posterior view of the anatomy of the greater occipital nerve, occipital artery, 3rd occipital nerve, lesser occipital nerve, greater auricular nerve, sternocleidomastoid muscle, and trapezius muscle. The occipital artery is included to illustrate its close relationship to the greater occipital nerve and its importance as a landmark for nerve localization while helping avoid inadvertent intravascular injection. Illustrated by: Zoë Rushetsky

**Fig. 11:**
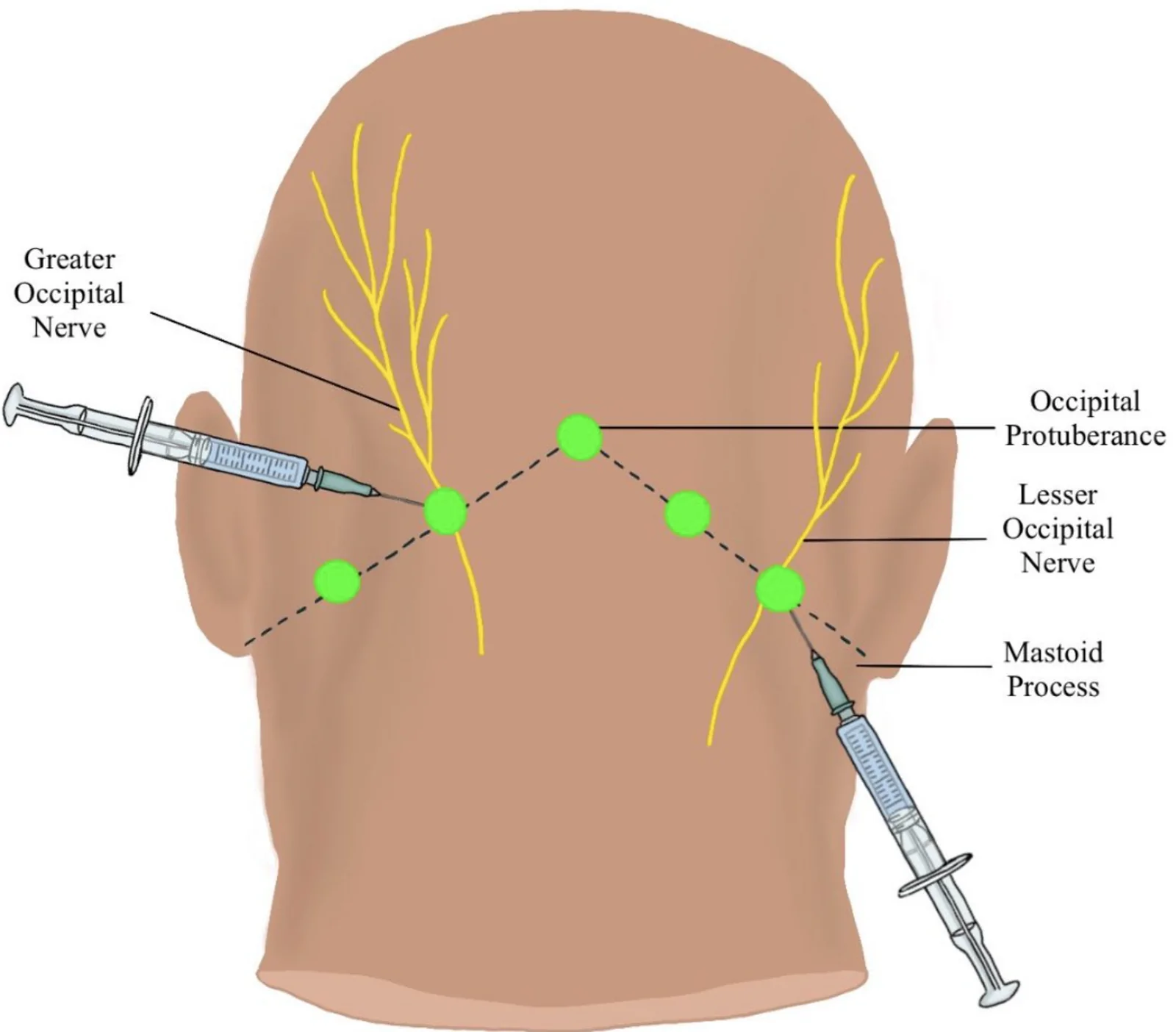
Injection sites for the greater occipital and lesser occipital nerve blocks. The medial needle indicates the greater occipital nerve block location. The lateral needle demonstrates local anesthetic infiltration along the superior nuchal line between the mastoid process and the external occipital protuberance to achieve blockade of the lesser occipital nerve. The external occipital protuberance and mastoid process are shown as key surface landmarks for localization. Illustrated by: Ambika Jokhulall

**Fig. 12:**
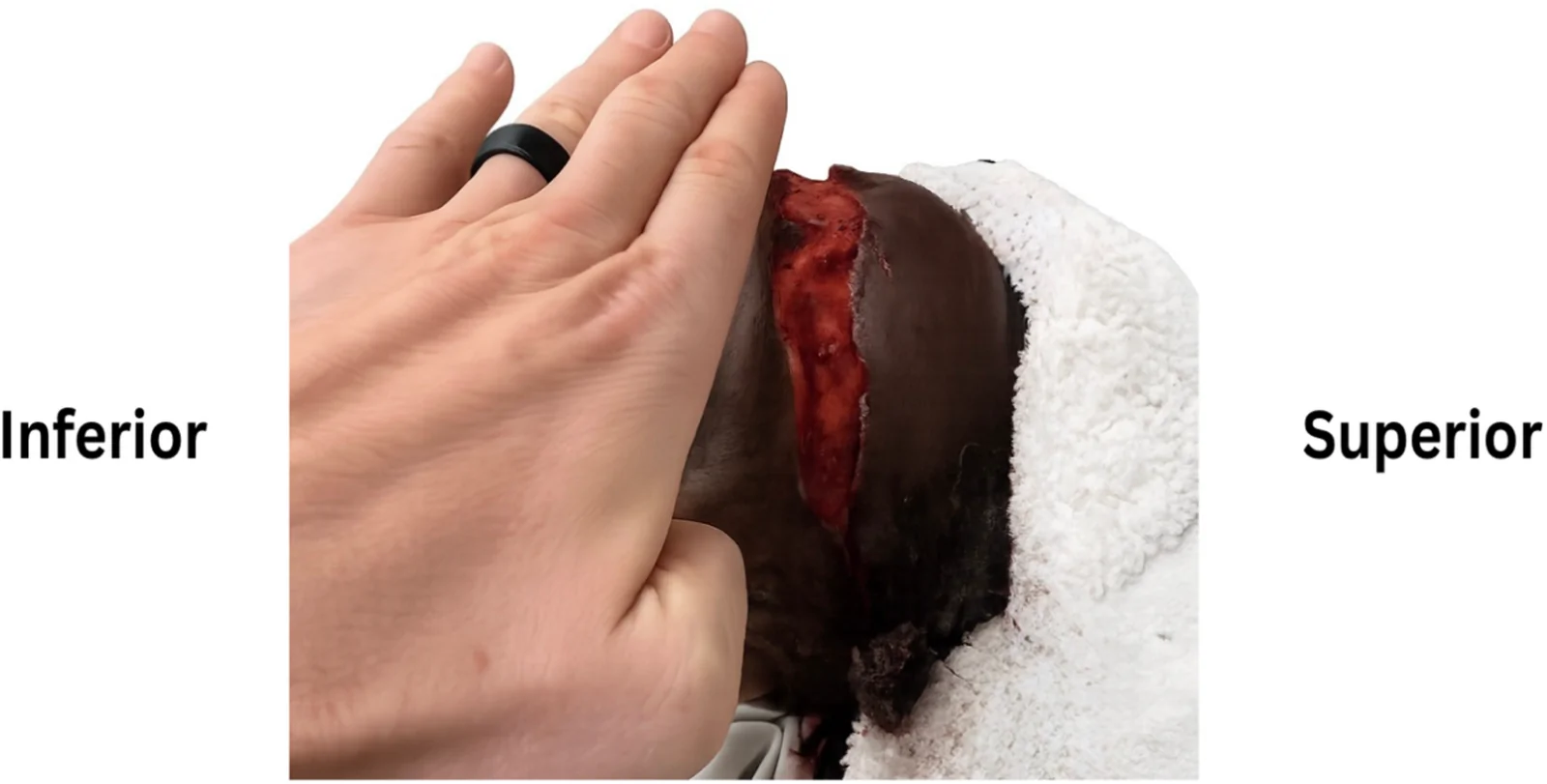
Acute occipital scalp laceration following trauma. The patient is positioned in the right lateral decubitus position, with the posterior scalp (occiput) facing the camera and the vertex oriented superiorly. The clinician’s hand is included for scale

The posterior anatomical course for these nerves is illustrated in Fig. 10, while injection sites for both the greater and lesser occipital nerves are shown in Fig. 11. A clinical example of an occipital scalp laceration amenable to regional injection of anesthesia is shown in Fig. 12.

Selection of posterior scalp nerve blocks should be guided by the location of the wound or planned procedure rather than routinely blocking all posterior scalp nerves. For example, isolated occipital lacerations typically require blockade of only the greater and/or lesser occipital nerves, whereas more extensive posterior scalp procedures may benefit from combined regional blockade.

These anatomical landmarks are reliable and enable emergency and urgent care clinicians to perform targeted nerve blocks efficiently and accurately, minimizing the need for sedation or extensive local anesthetic infiltration. Only the nerves supplying the affected region require blockade; comprehensive anesthesia is unnecessary unless a circumferential ring block is intended. For example, an isolated forehead laceration can typically be adequately anesthetized by blocking the supraorbital and supratrochlear nerves.

### Medication choices and the toxicity thresholds

The choice of local anesthetic plays an integral role in effectiveness as well as safety of head and neck nerve blocks in the urgent care setting. The most frequently used agents are lidocaine, bupivacaine, and ropivacaine [10], which all offer various roles in terms of onset, duration, and toxicity profile. A comparative summary of these agents is shown in Table 1.

**Table 1:** Regional anesthetics for head and neck nerve blocks

Medication	Onset (min)*	Duration (min)	Maximum dose without epinephrine (milligrams/kg)	Maximum dose with epinephrine (milligrams/kg)
Lidocaine (1–2%)	< 1	30–120	4	7
Bupivacaine (0.25–0.5%)	2–10	120–140	3	5
Ropivacaine (0.5%)	10–45	120–350	3	—

Adapted from Tintinalli’s Emergency Medicine [11]. *Onset times may vary

#### Precautions and adverse effects

These anesthetics are typically well-tolerated; however, caution should be exercised in patients with hepatic and cardiac impairment. Bupivacaine warrants caution in patients with underlying cardiovascular disease due to higher cardiotoxic potential [12]. All three anesthetics discussed are metabolized by the liver and should be used with caution in patients with hepatic impairment. Additional precautions should be taken in a patient who may present with abnormal anatomy, coagulopathy, known allergy to certain anesthetic agents, or have an infection present at the injection site.

Common adverse effects include hematoma at the site of injection and nerve injury. A hematoma can be prevented by applying ice to the site along with compression following injection. To reduce the risk of inadvertent intravascular injection, aspiration should be performed before each injection. More serious complications are manifestations of LAST and include agitation, confusion, tinnitus, perioral numbness, metallic taste, convulsions, respiratory depression, respiratory arrest, and cardiovascular collapse. Although maximum recommended doses are shown in Table 1, LAST may occur at substantially lower doses following inadvertent intravascular injection, particularly during head and neck nerve blocks [13–15]. Patients should be continuously monitored with a code cart nearby before, during, and after the procedure. LAST may present with agitation, confusion, dizziness, sleepiness, unusual sensations, tinnitus, perioral numbness, metallic taste, or difficulty speaking. If signs of LAST occur, intravenous 20% lipid emulsion, dosed based on ideal body weight, can be initiated to aid resuscitation once the patient is hemodynamically stable [16].

### Efficient and targeted anesthesia

One of the most important advantages of a regional scalp nerve block in urgent care is its ability to deliver efficient, targeted anesthesia while conserving time and resources. By anesthetizing the sensory nerves supplying the injured region, providers can achieve pain control with one or two precise injections rather than multiple wound infiltrations [17]. This may shorten procedure time and avoid tissue distortion which can potentially complicate wound approximation and affect cosmetic outcomes.

This approach also reduces the number of needle passes, minimizes total anesthetic volume, and improves patient comfort, particularly in pediatric patients who may become distressed by repeated injections [18]. An example is a forehead laceration, which can be repaired using only supraorbital and supratrochlear nerve blocks [5, 19]. Similarly, a patient with a posterior scalp injury may require only greater and lesser occipital nerve blocks [20]. By anesthetizing only the nerves supplying the injured region, the repair field is preserved without tissue distortion, and less anesthetic may be required than with direct wound infiltration.

For more extensive or irregular wounds, targeted nerve blocks may be combined with partial ring blocks to extend anesthetic coverage while remaining within safe dosing limits [21]. In pediatric patients, scalp nerve blocks may reduce pain and anxiety associated with repeated local anesthetic infiltration, while in older adults they may decrease the need for additional sedative medications and their associated risks [22].

### Limitations

Evidence supporting the use of regional scalp nerve blocks in emergency settings is derived largely from anesthesia, neurosurgical, and regional anesthesia literature, with comparatively limited randomized controlled trials conducted specifically in emergency department populations. Additionally, landmark identification may be challenging in patients with distorted anatomy, significant soft tissue swelling, prior surgical alterations, or extensive scar formation. Further prospective emergency department–based studies are warranted to evaluate workflow efficiency, patient satisfaction, and comparative outcomes relative to local infiltration techniques.

## Conclusion

Regional scalp nerve blocks provide a targeted, efficient, and patient-centered approach to anesthesia for head and neck injuries in urgent care. They maintain a clear operative field, reduce reliance on sedation, and improve the overall experience for both patients and clinicians. Given their simplicity, safety profile, and effectiveness, broader adoption of these techniques has the potential to elevate the standard of care and expand the procedural skill set within urgent care medicine.

## Data Availability

All data discussed in this review are derived from previously published sources cited in the reference list. Figures included in this manuscript were drawn by two of the authors.

## Abbreviations

ASRA: American society of regional anesthesia and pain medicine
C2–C3: Cervical spinal nerve roots 2 and 3
ED: Emergency department
LAST: Local anesthetic systemic toxicity
V1: Ophthalmic division of the trigeminal nerve
V2: Maxillary division of the trigeminal nerve
V3: Mandibular division of the trigeminal nerve
